# Identification of autophagy-related long non-coding RNAs in endometrial cancer via comprehensive bioinformatics analysis

**DOI:** 10.1186/s12905-022-01667-4

**Published:** 2022-03-23

**Authors:** Heng Liu, Yanxiang Cheng

**Affiliations:** 1 Department of Obstetrics and Gynecology, Huangpi District Renmin Hospital of Jianghan University, Wuhan, 430300 China; 2 Department of Obstetrics and Gynecology, Renmin Hospital of Wuhan University, Wuhan, 430060 China

**Keywords:** Endometrial cancer, Autophagy, Long non-coding RNAs, Bioinformatics analysis, Prognostic model, TCGA

## Abstract

**Background:**

Endometrial cancer is a common gynaecological malignancy with an increasing incidence. It is of great importance and value to uncover its effective and accurate prognostic indicators of disease outcomes.

**Methods:**

The sequencing data and clinical information of endometrial cancer patients in the TCGA database were downloaded, and autophagy-related genes in the human autophagy database were downloaded. R software was used to perform a Pearson correlation analysis on autophagy-related genes and long non-coding RNAs (lncRNAs) to screen autophagy-related lncRNAs. Next, univariate and multivariate Cox regression analyses were performed to select autophagy-related lncRNAs and construct the prognostic model. Finally, the accuracy of the prognostic prediction of the model was evaluated, the lncRNA–mRNA network was constructed and visualized by Cytoscape, and the gene expression profile of endometrial cancer patients was analysed by GSEA.

**Results:**

A total of 10 autophagy-related lncRNAs were screened to construct the prognostic model. The risk factors were AC084117.1, SOS1-IT1, AC019080.5, FIRRE and MCCC1-AS, and the protective factors were AC034236.2, POC1B-AS1, AC137630.1, AC083799.1 and AL133243.2. This prognostic model could independently predict the prognosis of endometrial cancer patients and had better predictive performance than that of using age and tumour grade. In addition, after classifying patients as high-risk or low-risk based on the prognostic model, we found that the enrichment of the JAK-STAT and MAPK pathways was significantly higher in the high-risk group than that in the low-risk group.

**Conclusions:**

The 10 autophagy-related lncRNAs are potential prognostic biomarkers. Compared with using age and tumour grade, this prognostic model is more predictive for the prognosis of endometrial cancer patients.

## Background

Uterine corpus carcinoma is one of the most common gynaecological malignancies in the world, and endometrial cancer accounts for most of it [1]. It is worth noting that the incidence of endometrial cancer in premenopausal and postmenopausal women has increased, and it is expected to increase further in the next ten years [2]. Endometrial cancer is characterized by a high degree of histological heterogeneity. Compared with patients with high-grade endometrioid, papillary serous, and clear cell histology, patients with low-grade endometrioid cancer usually have a better prognosis. This may be because high-grade endometrial cancer is more likely to appear in advanced stages and has a higher recurrence rate [3]. Patients who initially present with early endometrial cancer and experience recurrence have a lower survival rate, while patients who initially present with advanced (stage III/IV) endometrial cancer have a higher risk of recurrence and are more likely to develop extrapelvic metastases [4]. Therefore, finding effective prognostic markers for endometrial cancer is of great importance for improving the survival rate of patients with endometrial cancer and for improving the quality of life of patients.

Autophagy is a highly conserved process that isolates misfolded proteins and damaged or ageing organelles in autophagosome vesicles and transmits them to lysosomes for degradation [5–7]. Autophagy is a double-edged sword. It can be used as a tumour suppressor that selectively inhibits protein degradation and destroys organelles, and it can also be used as a cell survival promoter that promotes tumour growth [8–10]. Studies have shown that autophagy-related genes are frequently mutated in endometrial cancer, and that autophagy disorders are closely related to the occurrence and development of endometrial cancer [11–13]. Long non-coding RNAs (lncRNAs) are non-coding RNAs with a length of more than 200 nucleotides that can regulate gene expression at the transcriptional and posttranscriptional levels, and act as regulators of various pathophysiological conditions [14–16]. Studies have found that compared with those in normal tissues, lncRNAs in endometrial cancer have different expression. The imbalance of lncRNA expression is closely related to the grade of endometrial cancer, FIGO stage, depth of myometrial invasion, lymph node metastasis and patient survival rate [17–19]. A recent study showed that lncRNA HOTAIR can affect the resistance of endometrial cancer by regulating autophagy [20], which suggests that lncRNAs may participate in the progression of endometrial cancer by affecting autophagy, and that autophagy-related lncRNAs may be potential prognostic markers for endometrial cancer.

The aims of the study were to identify differentially expressed autophagy-related lncRNAs in endometrial cancer via a bioinformatics analysis and to construct a prognostic model. The autophagy-related lncRNAs involved in model construction may have important clinical significance for the prognostic prediction for endometrial cancer patients.

## Methods

### Data download and annotation

TCGA (The Cancer Genome Atlas; https://tcgadata.nci.nih.gov/tcga/) is a publicly funded project that aims to classify and discover cancer genome changes to create a comprehensive cancer genome atlas [21]. TCGA divides endometrial cancer into three disease types: “adenomas and adenocarcinomas”, “cystic, mucinous and serous neoplasms” and “epithelial neoplasms”, with 407, 139 and 2 cases, respectively, in the database. The transcriptome sequencing data of “adenomas and adenocarcinomas” and “cystic, mucinous and serous neoplasms” were downloaded for subsequent analysis. Clinical information for endometrial cancer patients was also downloaded from TCGA. The data were obtained from the public database in October 2020. The obtained data were differentiated into lncRNAs and mRNAs using Prel and R software. There was no need to obtain the approval of the ethics committee or the patient’s informed consent.

### Identification of autophagy-related lncRNAs

We downloaded 257 autophagy-related genes from the Human Autophagy Database (http://www.autophagy.lu/clustering/index.html) in October 2020 [22]. The mRNA expression quantification of these autophagy-related genes in endometrial cancer was extracted from the data downloaded from TCGA. Pearson correlation analysis was performed on the expression quantification of autophagy-related genes and lncRNAs using R software, and the correlation coefficients of |R| > 0.5 and *p* < 0.001 were used to screen autophagy-related lncRNAs.

### Construction of the prognostic model

Kaplan-Meier (KM) survival curves and univariate Cox regression analysis were used to identify the autophagy-related lncRNAs associated with patient prognosis. The overall survival (OS) of endometrial cancer patients was obtained from clinical information downloaded from TCGA. In the KM analysis, the patients were divided into two groups based on the median expression value of autophagy-related lncRNAs, and the OS of the two groups of patients was compared to determine whether there was a significant difference (*p* < 0.05). The hazard ratio (HR) was calculated and used to classify autophagy-related lncRNAs as risk factors (HR > 1) or protective factors (HR < 1). Next, univariate Cox regression analysis was performed to identify autophagy-related lncRNAs that were significantly correlated with the OS of endometrial cancer patients (*p* < 0.05). Autophagy-related lncRNAs with *p* < 0.05 in both KM and univariate Cox regression analyses were identified as autophagy-related lncRNAs associated with prognosis. Then, multivariate Cox regression analysis was performed on the identified autophagy-related lncRNAs associated with prognosis and the regression coefficient (*Coef*) of each autophagy-related lncRNA was obtained. Finally, the prognostic model consisting of 10 autophagy-related lncRNAs was obtained after optimization by the Akaike Information Criterion (AIC) value. The risk score was calculated for each patient based on the prognostic model as follows: Risk score = ${\sum }_{i=1}^{n}Coef\left(i\right)\times x\left(i\right)$, where *Coef* (*i*) and *x* (*i*) are the regression coefficient and expression level of autophagy-related lncRNAs used to construct the prognostic model, respectively.

### Evaluation of the prognostic model

The potential of the prognostic model as an independent prognostic factor was assessed by KM survival and ROC curve. The risk score of each endometrial cancer patient was calculated according to the prognostic model, and the median risk score was used as the demarcation point to divide endometrial cancer patients into high-risk and low-risk groups. The KM survival curve was used to compare the OS of the two groups of patients. The age and disease grade of endometrial cancer patients were obtained from clinical information downloaded from TCGA. The ROC curve was used to evaluate the prognostic effects of risk score, age, and tumour grade.

### Construction of the lncRNA–mRNA coexpression network

A coexpression network of autophagy-related genes and autophagy-related lncRNAs was constructed to visually observe their relationship. Cytoscape is a general open source software used for the large-scale integration of molecular interaction networks [23]. The relationship between the 10 autophagy-related lncRNAs identified above and the target autophagy-related genes (mRNA) was analysed, and Cytoscape (version 3.7.2) was used to construct and visualize the lncRNA–mRNA coexpression network.

### Gene set enrichment analysis

Gene set enrichment analysis (GSEA; http://www.broadinstitute.org/gsea) is a powerful analysis method that interprets gene expression data by focusing on gene sets [24]. The genome expression profiles of endometrial cancer patients were analysed by GSEA to determine the differentially expressed genes between the high-risk and low-risk patient groups, and significantly enriched gene sets were obtained, thereby identifying the signalling pathways that may be involved in the progression of the high-risk group.

### Statistical analysis

Perl (version 5.30.2) and R software (version 3.6.1) were used to process and analyse the data. Unless otherwise specified, *p* < 0.05 was considered statistically significant.

## Results

### Identification of autophagy-related lncRNAs with prognostic significance in endometrial cancer

RNA sequencing data of endometrial cancer patients were downloaded from the TCGA database, of which 14,142 lncRNAs and 19,658 mRNAs were identified. A total of 257 autophagy-related genes were extracted from the Human Autophagy Database. Pearson correlation analysis of lncRNAs and autophagy-related genes was conducted in R, and a total of 339 autophagy-related lncRNAs were identified using a correlation coefficient of |R| > 0.5 and *p* < 0.001 as the selection criteria.

**Fig. 1:**
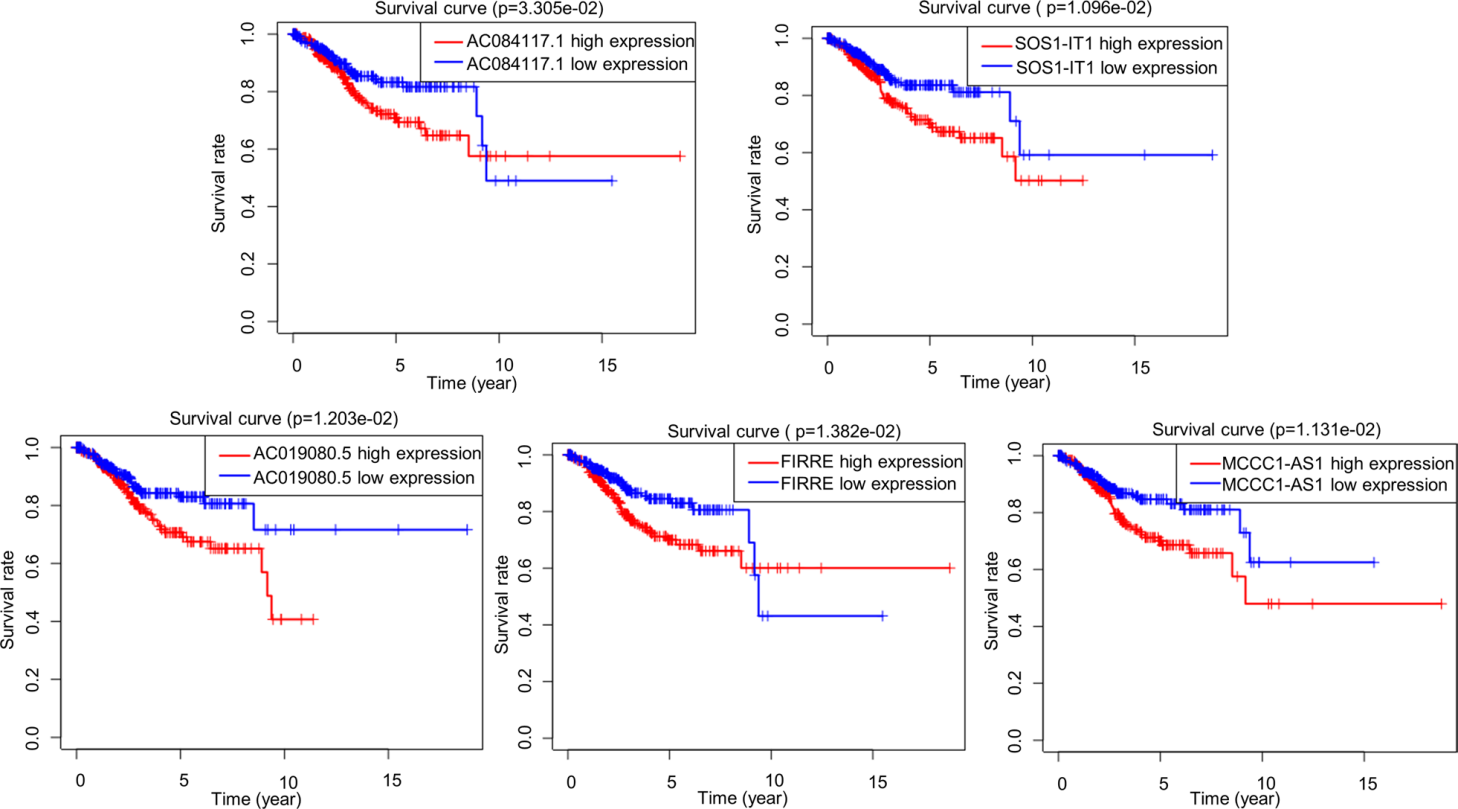
Survival curves of endometrial cancer patients grouped according to the expression levels of risk factors in the autophagy-related lncRNA prognostic model

**Fig. 2:**
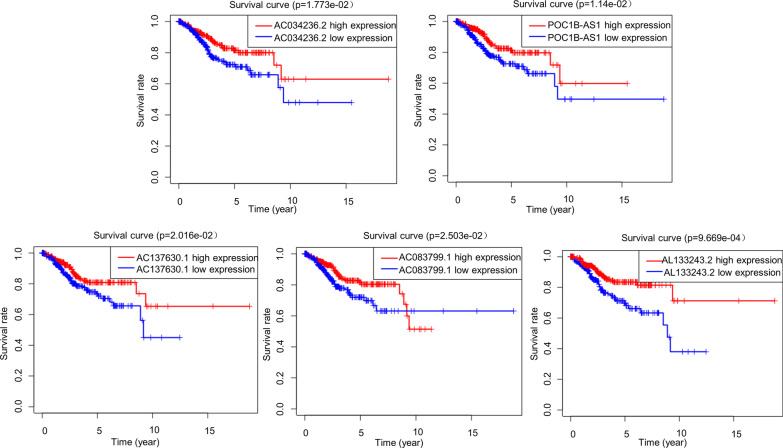
Survival curves of endometrial cancer patients grouped according to the expression levels of protective factors in the autophagy-related lncRNA prognostic model

Univariate Cox regression analysis was performed on the 339 autophagy-related lncRNAs, and it was found that the expression levels of 24 lncRNAs were associated with the OS of endometrial cancer patients (Table 1). Next, multivariate Cox regression analysis was performed on the 24 lncRNAs and optimized based on the AIC value. It was found that 10 of the 24 autophagy-related lncRNAs were good candidates for constructing the prognostic model (Table 2). Specifically, AC084117.1, SOS1-IT1, AC019080.5, FIRRE and MCCC1-AS1 were risk factors with HR > 1 (the OS in patients with a high autophagy-related lncRNA expression was significantly shorter than that in patients with a low expression) (Fig. 1). AC034236.2, POC1B-AS1, AC137630.1, AC083799.1 and AL133243.2 were protective factors with HR < 1 (the OS of patients with a high autophagy-related lncRNA expression was significantly longer than that of patients with a low expression) (Fig. 2).

**Table 1:** Autophagy-related lncRNAs significantly related to the OS of endometrial cancer patients

Autophagy-related lncRNAs	KM	B	SE	HR	HR.95 L	HR.95 H	*p*
AC017074.1	0.0014	0.0406	0.0173	1.0414	1.0068	1.0772	0.0188
AC084117.1	0.0331	0.1888	0.0961	1.2078	1.0004	1.4581	0.0495
TRAF3IP2-AS1	0.0085	0.4266	0.1964	1.5321	1.0426	2.2513	0.0298
AL163051.2	0.0137	0.0891	0.0390	1.0932	1.0127	1.1801	0.0224
AC026704.1	0.0309	− 0.3046	0.1227	0.7374	0.5798	0.9379	0.0130
AC034236.2	0.0177	− 0.3421	0.1521	0.7103	0.5272	0.9569	0.0245
POC1B-AS1	0.0114	− 1.0965	0.3925	0.3340	0.1548	0.7209	0.0052
ACBD3-AS1	0.0300	− 0.0715	0.0330	0.9310	0.8726	0.9932	0.0303
SOS1-IT1	0.0110	0.2480	0.0661	1.2814	1.1257	1.4587	0.0002
AC108134.4	0.0005	− 0.0810	0.0374	0.9222	0.8571	0.9923	0.0302
ELN-AS1	0.0458	− 0.0591	0.0270	0.9426	0.8941	0.9937	0.0283
AC137630.1	0.0202	− 0.8008	0.2787	0.4490	0.2600	0.7752	0.0041
NRAV	0.0091	− 0.0794	0.0273	0.9237	0.8756	0.9744	0.0036
AC019080.5	0.0120	0.4590	0.1801	1.5825	1.1119	2.2524	0.0108
LINC02166	0.0213	− 0.0790	0.0362	0.9241	0.8608	0.9920	0.0291
AC083799.1	0.0250	− 0.0331	0.0165	0.9675	0.9367	0.9992	0.0449
AL353622.1	0.0138	− 0.1189	0.0492	0.8879	0.8064	0.9777	0.0156
MUC20-OT1	0.0109	0.2544	0.1043	1.2897	1.0513	1.5823	0.0147
FIRRE	0.0138	0.2207	0.0614	1.2469	1.1055	1.4065	0.0003
Z98884.2	0.0253	0.2784	0.0975	1.3210	1.0912	1.5992	0.0043
ADNP-AS1	0.0094	0.1507	0.0760	1.1627	1.0017	1.3494	0.0474
AL133243.2	0.0010	0.1934	0.0772	1.2134	1.0431	1.4116	0.0122
AL353747.2	0.0007	− 0.2684	0.1032	0.7646	0.6246	0.9360	0.0093
MCCC1-AS1	0.0113	0.3904	0.1376	1.4776	1.1284	1.9350	0.0045

**Table 2:** Prognostic model constructed by 10 autophagy-related lncRNAs

Autophagy-related lncRNAs	coef	HR
AC084117.1	0.1963	1.2168
AC034236.2	− 0.2861	0.7512
POC1B-AS1	− 1.0096	0.3644
SOS1-IT1	0.1513	1.1633
AC137630.1	− 0.4837	0.6165
AC019080.5	0.6225	1.8636
AC083799.1	− 0.0256	0.9747
FIRRE	0.2143	1.2389
AL133243.2	− 0.2455	0.7823
MCCC1-AS1	0.2581	1.2945

### Evaluation of the prognostic model

According to the abovementioned prognostic model constructed from 10 autophagy-related lncRNAs, the risk score of each endometrial cancer patient in the TCGA database was calculated as follows: Risk score = (0.1963 × AC084117.1 expression level) + (− 0.2861 × AC034236.2 expression level) + (− 1.0096 × POC1B-AS1 expression level) + (0.1513 × SOS1-IT1 expression level) + (− 0.4837 × AC137630.1 expression level) + (0.6225 × AC019080.5 expression level) + (− 0.0256 × AC083799.1 expression level) + (0.2143 × FIRRE expression level) + (− 0.2455 × AL133243.2 expression level) + (0.2581 × the expression level of MCCC1-AS1).

**Fig. 3:**
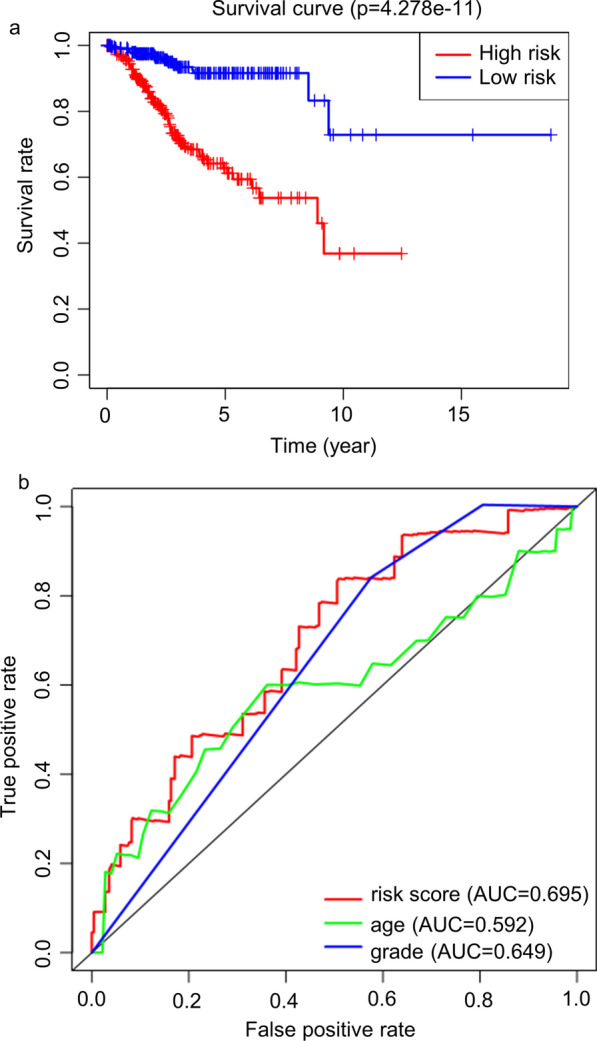
The prognostic model constructed by autophagy-related lncRNAs is an independent prognostic factor for patients with endometrial cancer. **a** KM survival curve. The red curve is the survival curve of the high-risk group, and the blue curve is the survival curve of the low-risk group. **b** ROC curve of the autophagy-related lncRNA prognostic model

**Fig. 4:**
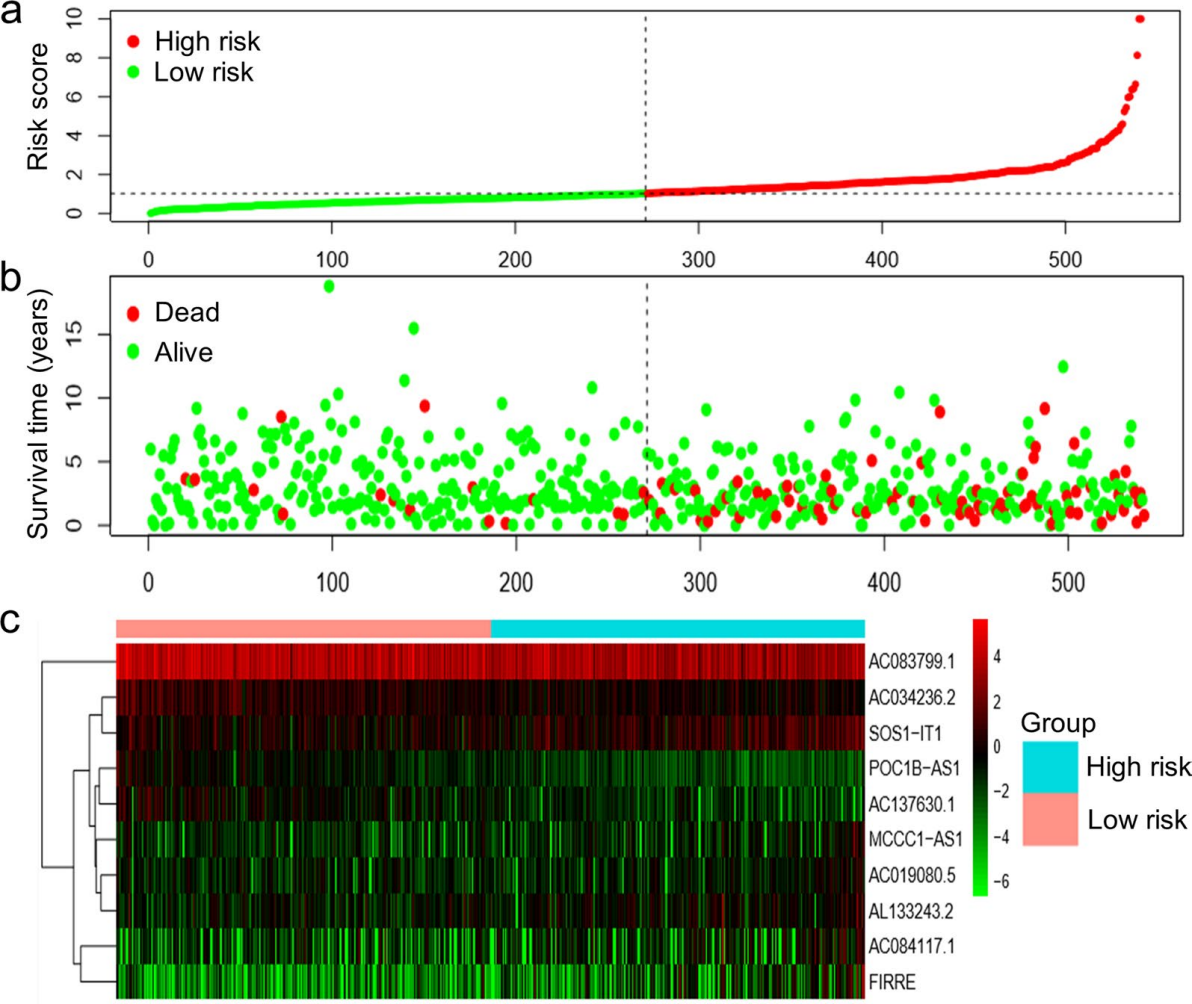
Risk curves of endometrial cancer patients with high and low risk scores. **a** Risk scores for patients with endometrial cancer. Red points indicate endometrial cancer patients with high risk scores, and green points indicate endometrial cancer patients with low risk scores. **b** Survival time of endometrial cancer patients in the high-risk and low-risk groups. The vertical dotted line separates the high-risk group (right side) and the low-risk group (left side). The red dots indicate patients who died, and the green dots indicate surviving patients. **c** Heatmap of the expression levels of 10 autophagy-related lncRNAs in the high-risk group and the low-risk group

According to the median risk score, patients with endometrial cancer were divided into high-risk and low-risk groups. The KM survival curve showed that the OS of endometrial cancer patients in the high-risk group was significantly shorter than that of endometrial cancer patients in the low-risk group (Fig. 3a). The five-year survival rate of the high-risk group was 61.3%, and the 5-year survival rate of the low-risk group was 91.6%. The area under the ROC curve (AUC value) of the risk score was 0.695, which was higher than the AUC value of age (AUC = 0.592) and tumour grade (AUC = 0.649) (Fig. 3b), indicating that the prognostic model was an independent prognostic factor for patients with endometrial cancer. The endometrial cancer patients were ranked according to the risk score calculated by the autophagy-related lncRNA prognostic model (Fig. 4a). Patients with higher risk scores had shorter survival times (Fig. 4b). There were significant differences in the expression levels of 10 autophagy-related lncRNAs as prognostic marker molecules in high-risk and low-risk endometrial cancer patients (Fig. 4c). The high-risk group had higher expression levels of risk factors (AC084117.1, SOS1- IT1, AC019080.5, FIRRE and MCCC1-AS1), and the low-risk group had higher expression levels of protective factors (AC034236.2, POC1B-AS1, AC137630.1, AC083799.1 and AL133243.2).

### Construction of the lncRNA–mRNA coexpression network and GSEA

**Fig. 5:**
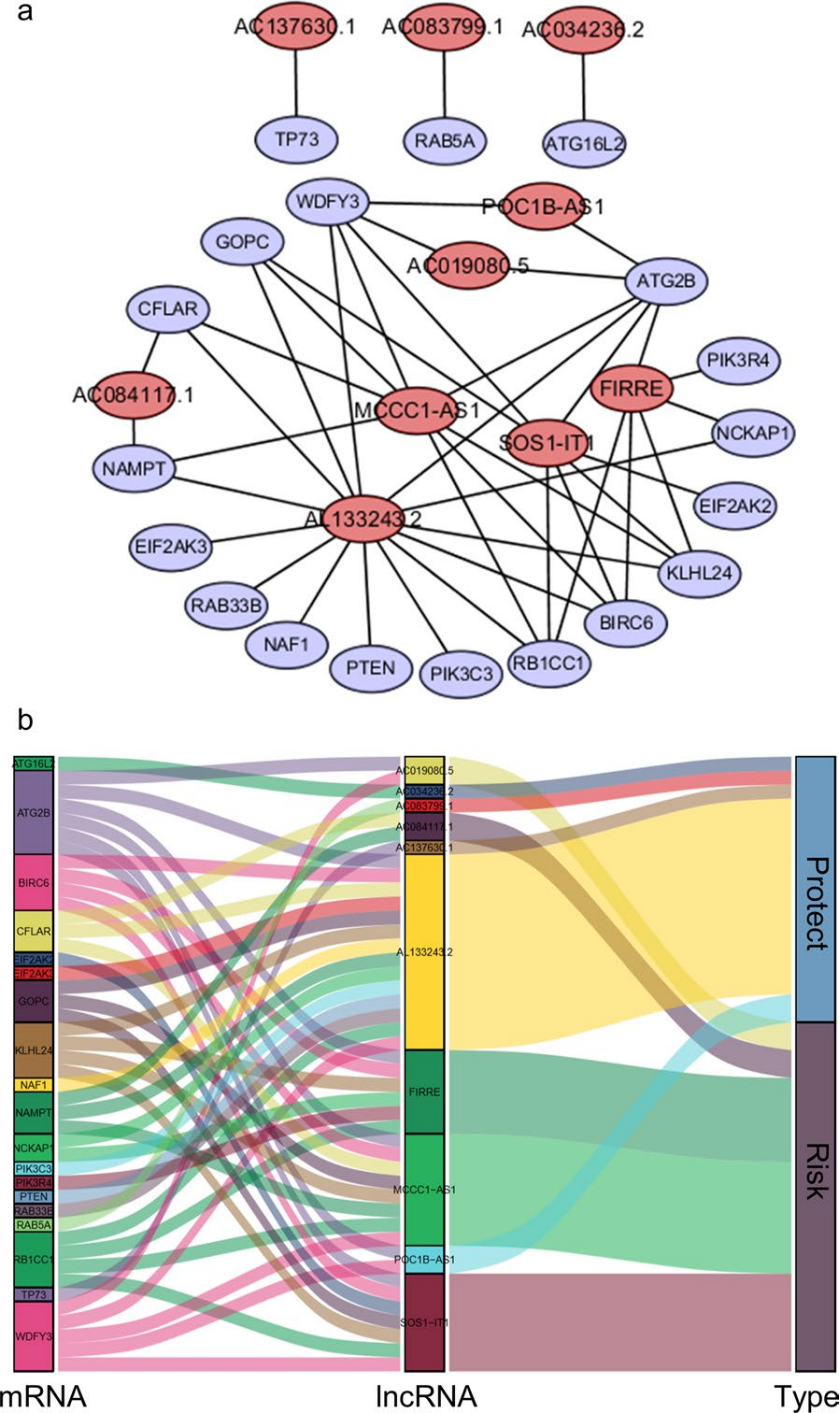
LncRNA–mRNA coexpression network. **a** Pink circles indicate 10 autophagy-related lncRNAs that are closely related to the overall survival of endometrial cancer patients identified by multivariate Cox regression analysis, and purple circles indicate autophagy-related genes coexpressed with autophagy-related lncRNAs. **b** The relationship between mRNA and autophagy-related lncRNAs

**Fig. 6:**
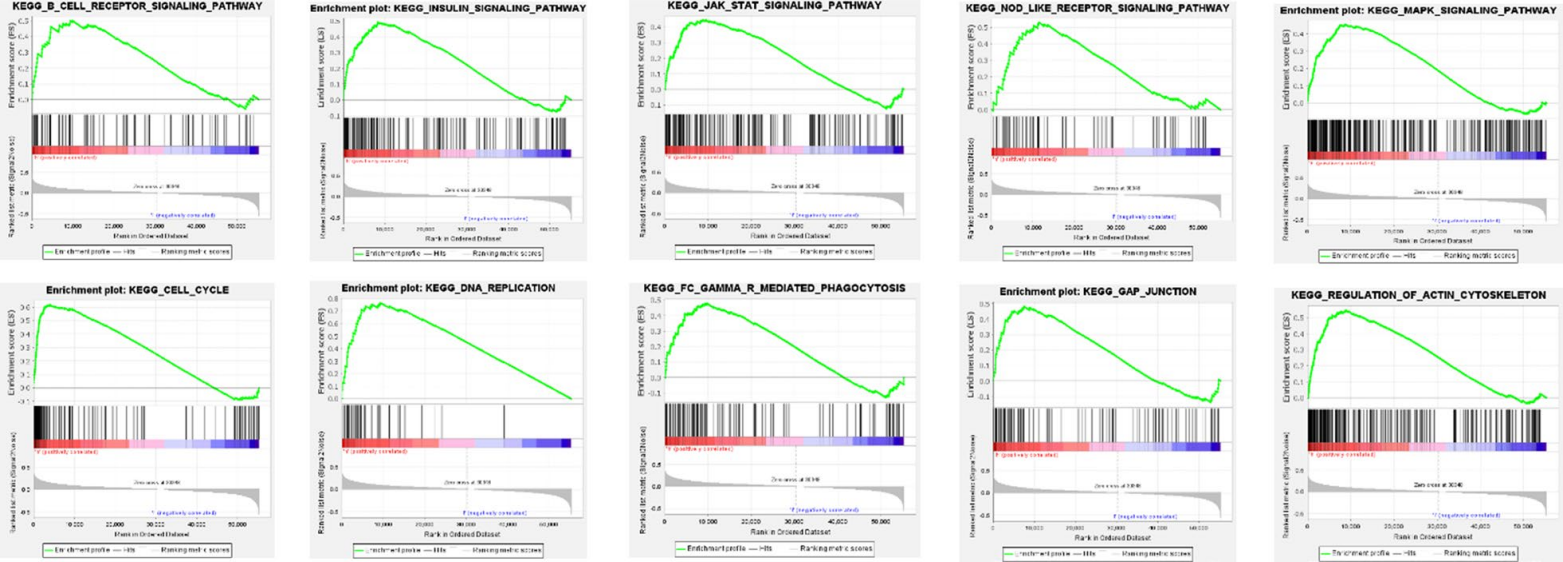
Gene set enrichment analysis of high-risk and low-risk endometrial cancer patients based on autophagy-related lncRNA prognostic models

Cytoscape was used to construct a lncRNA–mRNA coexpression network to study the potential functions of 10 autophagy-related lncRNAs in endometrial cancer. The lncRNA–mRNA coexpression network contained 44 pairs of lncRNA–mRNAs screened by the Pearson correlation coefficient |R| > 0.5, *p* < 0.001 (Fig. 5a). A Sankey diagram showed the relationship between 19 mRNAs and 10 lncRNAs (risk factors and protective factors) (Fig. 5b). In addition, the GSEA results showed that the genetic changes in the high-risk endometrial cancer patient group were related to the B cell receptor, insulin, JAK-STAT, MAPK and NOD-like receptor signalling pathways. Cell cycle, DNA replication, Fcγ receptor-mediated phagocytosis, gap junctions, and regulation of actin cytoskeleton were also significantly increased in the high-risk endometrial cancer patient group (Fig. 6). These results provide a useful reference for the individualized treatment of endometrial cancer patients classified to different risk score groups in the future.

## Discussion

Patients with advanced endometrial cancer (FIGO stage III or IV) are at risk of local and systemic recurrence. Factors such as histological subtypes, degree of lymph node involvement, and completeness of surgical resection are useful indicators for predicting the prognosis of patients with endometrial cancer [25, 26]. However, there is still a lack of reliable and specific molecular markers for the prognosis of endometrial cancer. With the rapid development of sequencing methods, high-throughput biotechnology has been increasingly used in tumour diagnosis and prognosis prediction [27–29]. In this study, we analysed the differentially expressed autophagy-related lncRNAs in endometrial cancer patients via bioinformatics analysis, and constructed a prognostic model. On the one hand, the prognostic model can predict the prognosis of endometrial cancer patients from a molecular perspective. Focusing on or shortening the follow-up interval for high-risk patients may be an effective clinical intervention. On the other hand, the expression levels of these autophagy-related lncRNAs used to construct the prognostic model were different between the high-risk group and the low-risk group, and the enrichment of signalling pathways was also different between the two groups, which may provide a rationale for uncovering the molecular mechanisms of endometrial cancer progression and for developing new targeted therapies.

We downloaded the clinical information and sequencing data of endometrial cancer patients in TCGA and downloaded autophagy-related genes from the Human Autophagy Database. A total of 339 autophagy-related lncRNAs differentially expressed in endometrial cancer were identified with Perl and R software. Next, univariate and multivariate Cox regression analyses were performed on the identified 339 autophagy-related lncRNAs, and a total of 10 autophagy-related lncRNAs were finally used to construct the prognostic model. Endometrial cancer patients were divided into high-risk and low-risk groups according to the risk score calculated by the prognostic model. The KM survival curve showed that the OS of endometrial cancer patients in the low-risk group was significantly longer than that in the high-risk group. In addition, the area under the ROC curve indicated that the risk score calculated by the prognostic model was an independent prognostic factor that was superior to that calculated by using age and tumour grade. These results indicated that the autophagy-related lncRNA prognostic model we constructed was an independent factor significantly related to the OS of patients with endometrial cancer.

Previous clinical, biological and epidemiological studies have shown that the excessive and/or prolonged exposure to nonresistant oestrogen can increase the risk of endometrial cancer [30, 31]. Studies have shown that oestrogen can promote the viability of oestrogen-sensitive endometrial cancer cells and inhibit their autophagy levels [9], and that the oestrogen-inducing gene *EIG121* can regulate autophagy and promote the survival of endometrial cancer cells [32]. In addition, Ayabe et al. [33] found that the expression of glucagon-like peptide-1 receptor (GLP-1R) in endometrial cancer tissues was related to oestrogen receptor and progesterone receptor. GLP-1R agonists could stimulate autophagy and induce apoptosis of endometrial cancer cells via the AMPK pathway. These studies indicated that autophagy dysregulation may be a key factor in the occurrence and development of endometrial cancer. The 10 autophagy-related lncRNAs that we identified all directly or indirectly regulate autophagy, suggesting that these 10 lncRNAs may play an important role in endometrial cancer, and they may be potential therapeutic targets for the treatment of endometrial cancer.

Among the 10 autophagy-related lncRNAs, AC084117.1, SOS1-IT1, AC019080.5, FIRRE and MCCC1-AS1 were considered to be risk factors with an HR > 1, while AC034236.2, POC1B-AS1, AC137630.1, AC083799.1 and AL133243.2 were considered to be protective factors with an HR < 1. A recent study of the molecular biomarkers of multiple RNA types in endometrial cancer found that MCCC1-AS1 may regulate endometrial cancer by targeting *TNIK*. MCCC1-AS1 may be a new predictor of endometrial cancer recurrence [34]. This is consistent with the results of our research. The remaining 9 lncRNAs have not been previously studied and provide new directions for future research.

The prognostic model consisting of 10 autophagy-related lncRNAs can classify endometrial cancer patients into high-risk and low-risk groups. The significantly enriched signalling pathways between the two groups were different, which provides a basis for revealing the molecular mechanism of endometrial cancer progression. In addition, compared with age and disease grade, the risk score calculated according to the prognostic model can better predict the prognosis of patients, indicating that the prognostic model has good potential for predicting the prognosis of endometrial cancer patients. However, this study also has some limitations. For example, the expression levels of these autophagy-related lncRNAs have not been validated in endometrial cancer tissues, and validation of this prognostic model at the clinical level is also lacking. We hope to collect the clinical information and endometrial cancer tissues of patients to further verify the clinical application value of this prognostic model in the future.

## Conclusions

In the current study, we analysed and identified 10 autophagy-related lncRNAs via bioinformatics analysis, and constructed a prognostic model. They are significantly related to the OS of endometrial cancer patients and are potential prognostic biomarkers.

## Data Availability

The datasets analysed in the current study are available in TCGA (The Cancer Genome Atlas; https://tcgadata.nci.nih.gov/tcga/).

## Abbreviations

lncRNA: Long non-coding RNA
TCGA: The Cancer Genome Atlas
OS: Overall survival
HR: Hazard ratio
AIC: Akaike information criterion
KM: Kaplan–Meier
GSEA: Gene set enrichment analysis
GLP-1R: Glucagon-like peptide-1 receptor
